# Prediction of Bone Formation Rate of Artificial Bone With Machine Learning Models Considering the Variation of Experimental Results

**DOI:** 10.1002/ansa.70021

**Published:** 2025-06-09

**Authors:** Yuta Sakai, Shota Horikawa, Mamoru Aizawa, Hiromasa Kaneko

**Affiliations:** ^1^ Department of Applied Chemistry School of Science and Technology Meiji University, Tama‐ku Kawasaki Kanagawa Japan

**Keywords:** artificial bone, biomaterial, machine learning

## Abstract

The proportion of older people in the world's total population is expected to increase. Bone diseases are more prevalent in older people; therefore, the number of patients with such diseases is expected to increase worldwide. Artificial bone is a biomaterial used in the treatment of bone diseases. Artificial bones with high bone formation rates are desired; however, the results of artificial bone implantation vary. There are also ethical issues associated with animal experiments. Our purpose in this study is to predict the variation in bone formation rates. We created multiple sub‐datasets and constructed a machine learning model to predict the variation in bone formation rates by considering the results of multiple measurements. We also propose a metric, Jensen–Shannon (JS) divergence, to evaluate the accuracy of the model for predicting variation. We tested the validity of JS divergence by comparing combinations of explanatory variables. Additionally, we found an optimal combination of explanatory variables to construct a model with high predictive accuracy. We expect that the prediction of variation will be useful for improving the practical development of materials and medicines, such as artificial bones, for which stable effects are required, regardless of the individual.

## 1 Introduction

The proportion of older people in the total population of the world has risen from 5.1% in 1950 to 9.3% in 2020, and is expected to continue to rise [1]. With the extension of the average lifespan, it is predicted that millions of people will suffer from bone‐affecting diseases in many countries [2]. Therefore, it is expected that the number of bone diseases, such as bone loss, will increase worldwide.

Most therapies for bone defects are based on an autograft or allograft; however, these therapies have disadvantages, such as limited sources of supply and the risk of immune reactions. Thus, artificial bone, particularly types that promote bone regeneration, is currently the focus of attention [3]. For example, hydroxyapatite (Ca_10_(PO_4_)_6_(OH)_2_; HAp) and β‐tricalcium phosphate (β‐Ca_3_(PO_4_)_2_; β‐TCP) are expected to be useful in dentistry and orthopaedics because their structures are similar to human bone tissue [4]. Researchers have shown that highly biologically active materials can be synthesised by incorporating silicon, which is necessary for bone growth [5]. The optimisation of the physicochemical parameters of artificial bone [6] is also being researched. HAp ceramics are currently used as artificial bone in clinical settings; however, autologous bone grafting achieves better results than artificial bone grafting [7].

The bone formation rate is one of the most important properties for artificial bone and is measured using implantation in animals. Such animal experiments are necessary for the artificial bone development; however, two problems exist: first, the bone formation rate in experimental animals cannot be measured until months after implantation, and second, animal sacrifice should be avoided from an ethical point of view [8]. To address these issues, in a previous study, researchers constructed a two‐stage machine learning model to predict the bone formation rate of artificial bone [9]. The first model predicted physical properties from the experimental conditions, and the second model predicted the bone formation rate of artificial bone from spectral data and the physical properties predicted using the first model. In the dataset used in the previous study [9] with machine learning, multiple results for the bone formation rate existed for each sample or artificial bone because the implantation experiments were conducted on multiple individuals. The average values of the multiple results for the bone formation rate were processed as representative values and machine learning models were constructed. Although the models can predict the bone formation rate, they cannot predict how the bone conformation rate will vary for each artificial bone. Whilst the high bone formation rate is crucial in evaluating artificial bone, it is equally important that the results of the bone formation rate are stable, meaning that there is little variation. Therefore, it is a problem in the design of artificial bones to predict only the average of the bone formation rate of artificial bone. Additionally, no method exists for evaluating the predictive accuracy of a model when there are multiple measured values and estimated values.

Therefore, our objective is to develop a machine learning framework capable of predicting not only the average but also the variation in the bone formation rate from artificial bones, using ensemble modelling and distributional similarity metrics. This approach seeks to support the design of reproducible biomaterials and reduce reliance on animal testing. We prepare numerous sub‐datasets by randomly sampling from a dataset, and constructed a regression model for each sub‐dataset. When 100 models are constructed, for example, the constructed 100 models output 100 values of the bone formation rate for each sample, which makes it possible to represent the distribution of the bone formation rate. Additionally, we propose a metric based on Jensen–Shannon (JS) divergence for quantitatively comparing distributions.

## 2 Novelty of this work

A sub‐dataset‐based ensemble prediction strategy was developed to model and quantify variability in the bone formation rate of artificial bone, which has not been addressed in prior studies.

A new application of JS divergence was proposed to evaluate the predictive performance for distributional outputs—offering a complementary metric to conventional *r* ^2^.

The proposed method was applied in the biomaterials field, particularly for artificial bone, where animal‐to‐animal variability poses a serious issue for ethical and practical experimentation.

## 3 Methods

### 3.1 Dataset

**FIGURE 1:**
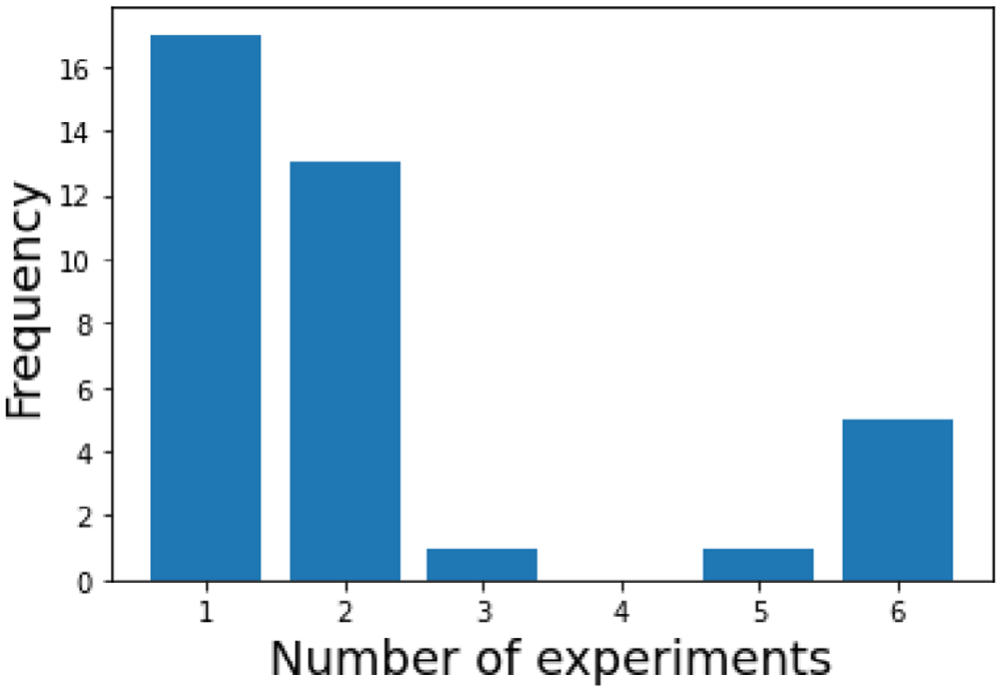
Histogram of the number of experiments.

**FIGURE 2:**
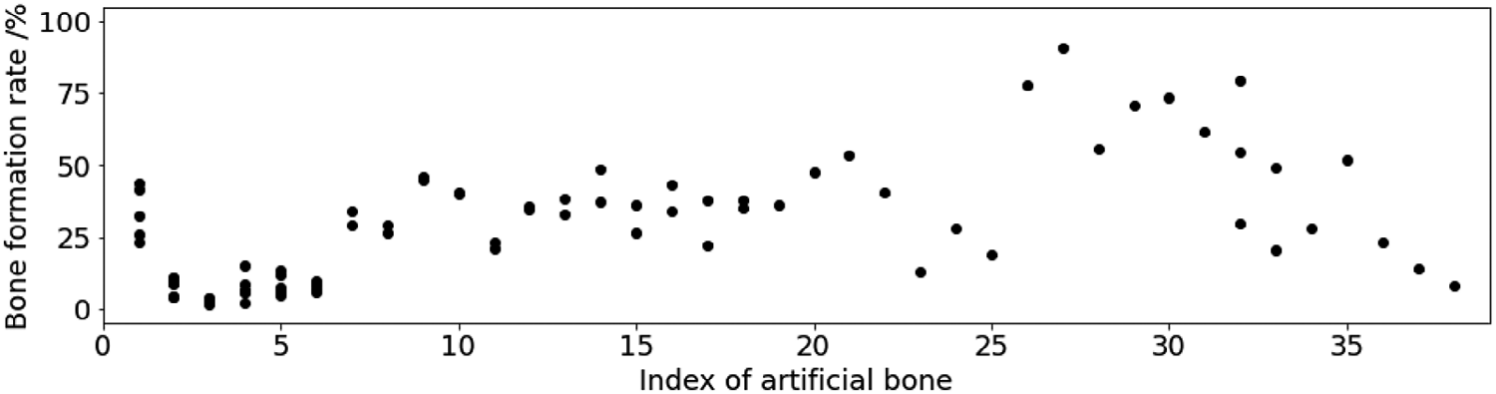
Measured bone formation rates for each artificial bone material.

Our dataset contained 38 types of artificial bone synthesised using two methods: the wet process [7] and homogeneous precipitation method [10]. The features were experimental conditions, animal experimental conditions, material properties, Fourier transform infrared spectroscopy (FT‐IR) and X‐ray diffraction (XRD) spectral data, and scanning electron microscope (SEM) images. Experimental conditions include the amount of reagents used in synthesis, the heating temperature, the amount of carbon beads added, as well as the animals used in animal experiments and the time required. Material properties include variables that are measured in a relatively short time, such as relative density and compressive strength. These are properties that are known to affect bone formation when controlled [11, 12]. FT‐IR and XRD are used for the identification of functional groups and crystalline phases [13]. Additionally, SEM images are often employed for analysing the structure of artificial bone [14]. There were one or several results for the bone formation rate for each artificial bone because we conducted the implantation experiments on multiple individuals. As shown in Figure 1, 17 types of artificial bone were conducted only one animal experiment and the others were conducted multiple animal experiments. Additionally, as shown in Figure 2, several bone formation rates for each artificial bone varied.

### 3.2 Sub‐Datasets

When there were multiple values in an objective variable *y* for each sample, we typically used the average value of *y* in the general model construction. However, a model that considers variations of *y* cannot be constructed with average values. Therefore, we prepared many sub‐datasets by randomly selecting one *y* value from each sample. In these sub‐datasets, the explanatory variables *X* were the same for each sample.

### 3.3 Model Construction

We constructed a machine learning model between *X* and *y* for each sub‐dataset, which resulted in the same number of models as sub‐datasets. When constructing the model, we considered multiple regression methods and selected the best method for each sub‐dataset. Inputting values of *X* for a sample into these models output the same number of estimated values as the model, which allowed us to predict the variability of *y*.

### 3.4 Jensen–Shannon Divergence

The sample had multiple measured values and multiple estimated values. The closer the distance between the distributions of the measured values and estimated values, the better the prediction results. Therefore, we introduced JS divergence as a metric for the distance between distributions. JS divergence is calculated from the following two equations:

(1) $$D_{\mathrm{KL}}\;\left(P∥Q\right)=\int_{x}p\left(x\right)\log\frac{p\left(x\right)}{q\left(x\right)}dx\;$$

(2) $$D_{\mathrm{JS}}\;\left(P∥Q\right)=\frac{D_{\mathrm{KL}}\left(P∥\frac{P+Q}{2}\right)+D_{\mathrm{KL}}\left(Q∥\frac{P+Q}{2}\right)}{2},\;$$

where *p*(*x*) and *q*(*x*) are probability distributions, *D*_KL_ denotes Kullback–Leibler divergence, and *D*_JS_ denotes JS divergence. Equation (1) is the formula for calculating *D*_KL_, which calculates the similarity of probability distributions *p*(*x*) and *q*(*x*). Equation (2) is the formula for calculating *D*_JS_ that gives symmetry to *D*_KL_ by making *p*(*x*) and *q*(*x*) interchangeable. The value of this metric is greater than or equal to 0. The closer it is to 0, the closer the distance between the two probability distributions. To evaluate the results of model construction for this indicator, we assumed that the actual and estimated values of the sample followed a normal distribution.

## 4 Results and Discussion

We used artificial bone synthesis conditions, animal experimental conditions, and physical properties for all model construction. We used eight combinations of three types of variables, FT‐IR, XRD, and SEM images, as X in methods A–H. As shown in Table 1, in each of the methods, variables with 〇 were used and variables with × were not used in the modelling of *X*.

**TABLE 1:** Methods and combinations of variables.

Method	FT‐IR	XRD	SEM	*D*_JS_	*r^2^*
A	×	×	×	0.726	−0.496
B	〇	×	×	0.370	0.579
C	×	〇	×	0.383	0.500
D	×	×	〇	0.707	−0.501
E	〇	〇	×	0.298	0.562
F	〇	×	〇	0.334	0.439
G	×	〇	〇	0.383	0.500
H	〇	〇	〇	0.361	0.537

Abbreviations: FT‐IR, Fourier transform infrared spectroscopy; XRD, X‐ray diffraction; SEM, scanning electron microscope.

**FIGURE 3:**
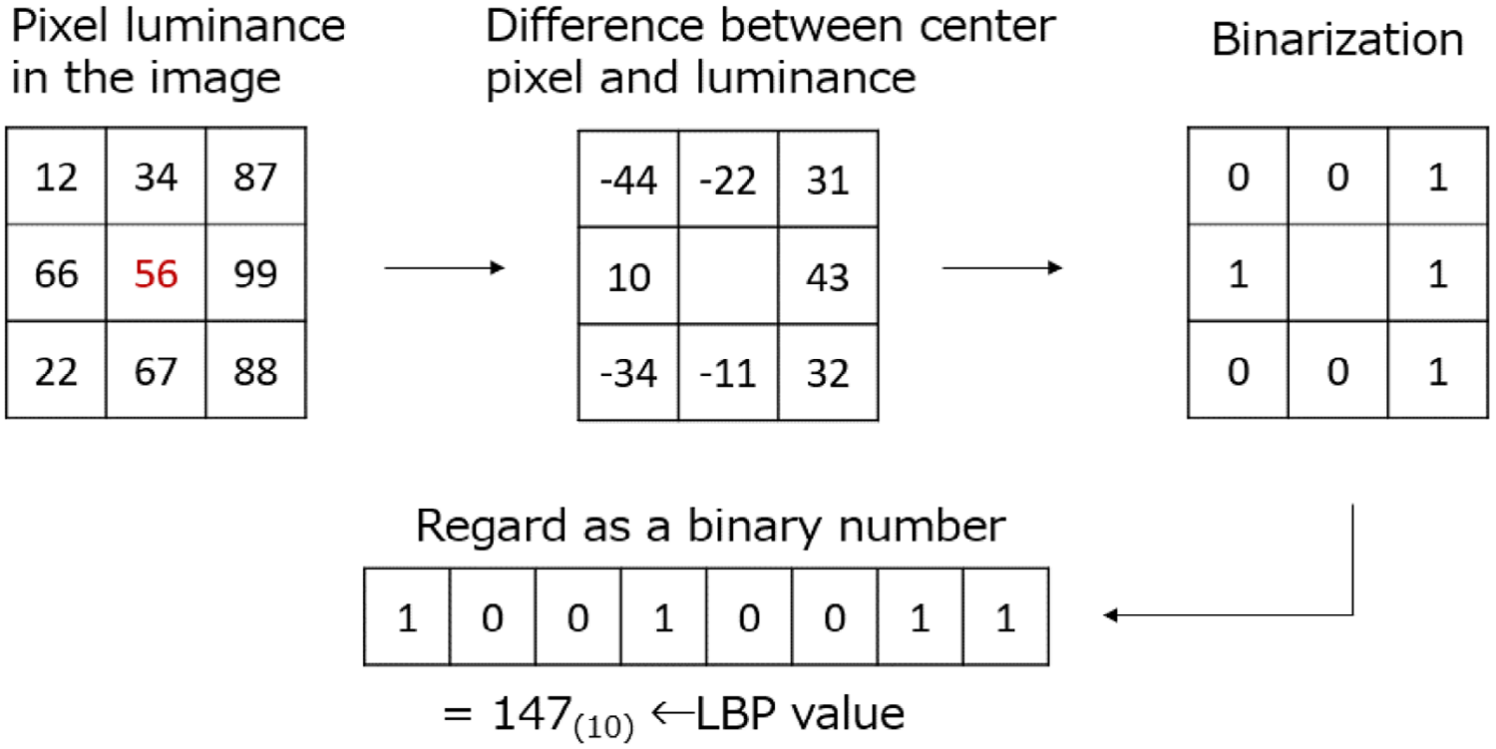
Overview of the LBP. LBP, local binary pattern.

For FT‐IR and XRD spectra, we selected wavenumbers and diffraction angles related to HAp and β‐TCP, which we used as materials, and used the intensity at those wavenumbers as *X*. For SEM images, we converted the brightness at each image pixel to a local binary pattern (LBP) and used the frequency of the value as the feature value. Additionally, we converted the luminance at each image pixel to an LBP and used the frequency of the value as the feature. As shown in Figure 3, for a given pixel, we calculated the difference in luminance from the surrounding pixels. We binarised these values so that they were 0 if they were negative and 1 if they were positive. We considered the flattened binary pixel as a binary number. We converted this binary number to a decimal number and considered it as the LBP value for the pixel. Because the LBP uses relative values, it is more sensitive to changes in the shading of the image than luminance.

We set the number of sub‐datasets to 100 and constructed a model for each sub‐dataset. The regression analysis methods we used in model construction were ordinary least squares [15], partial least squares regression [16], ridge regression [17], least absolute shrinkage and selection operator [17], elastic net [17], linear support vector regression [18]for the linear regression method and Gaussian process regression [19], nonlinear support vector regression [18], decision tree [20], random forest [21], gradient boosting decision tree [22], extreme gradient boosting [23], and light gradient boosting model [23] for the nonlinear regression method. We evaluated the predictive ability of each model using double cross‐validation [24] (inner fold: 5, outer fold: 10). We calculated *D*_JS_ for each material assuming that the bone formation rate of artificial bone followed a normal distribution. For samples with one or two measured values for the bone formation rate, we substituted the standard deviation for calculating the distribution for the average of the standard deviations of samples with three or more measured values.

Method A achieved the worst accuracy in *D*_JS_ and second worst in *r^2^* compared with all other methods. Methods B and C improved accuracy over method A by adding the spectra variables. By contrast, method D, which used SEM image features, did not achieve a significant improvement in accuracy. This may be because we used iterative Gaussian mixture regression [23] to fill in missing variables in the SEM images, but its accuracy was not very high. Combining multiple types of variables resulted in an improvement in accuracy for all methods. Method E had the best values for both *D*_JS_ and *r^2^*. Compared with Method E, Method F had a similarly superior *D*_JS_ and inferior *r^2^*. Method B had a superior *r^2^* but inferior *D*_JS_. Furthermore, Method D had worse values for both metrics. We compared four methods, that is, E, F, B and D with different trends in the two metrics, to confirm the validity of *D*_JS_ as an evaluation metric.

**FIGURE 4:**
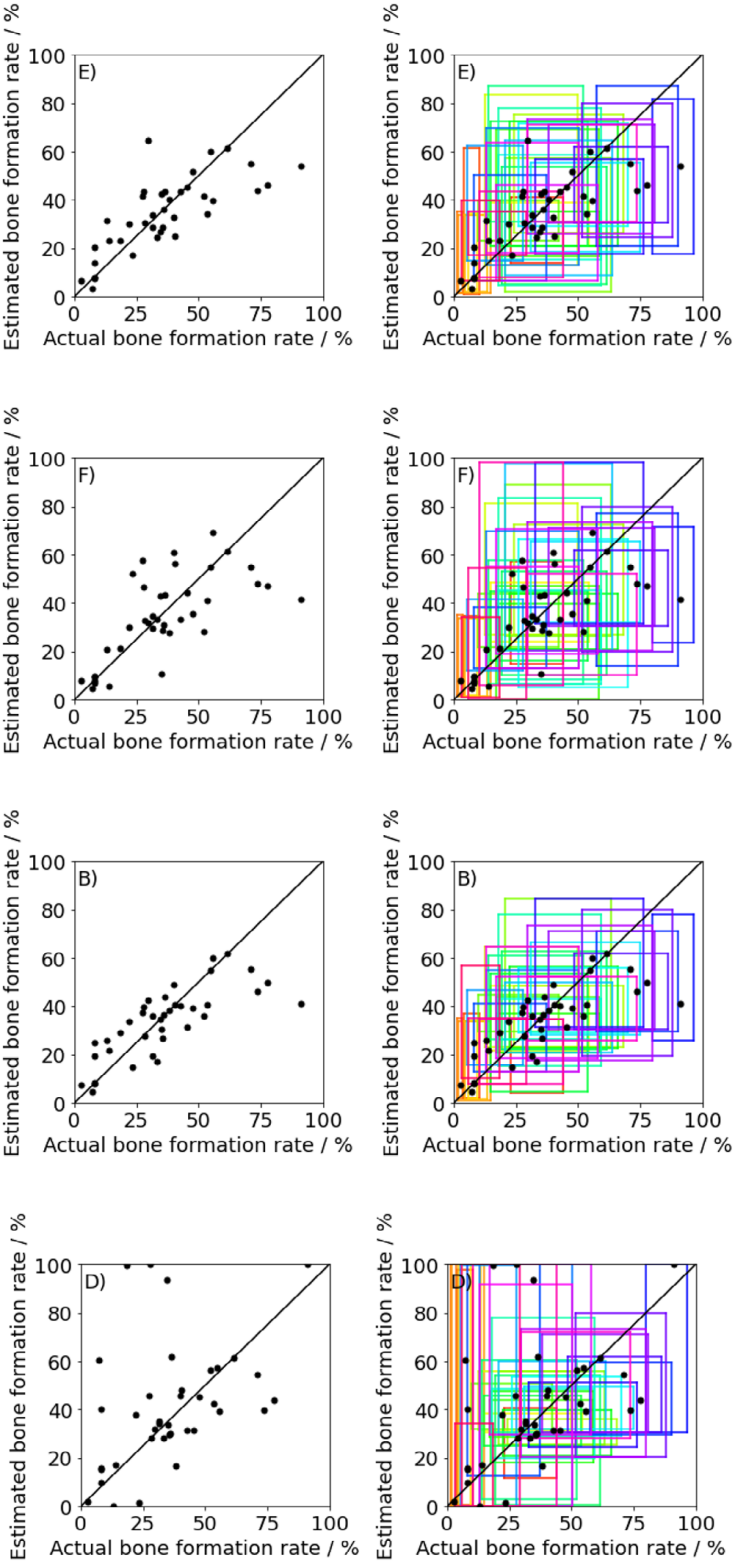
Scatter plots of each average of the predicted and measured bone formation rates with rectangles showing the dispersion.

The plots of the average of the measured and estimated values of methods B, D, E and F are shown in Figure 4, and the other plots are shown in Figure S1, with rectangles added to the plot with the horizontal length as the variation in the measured values and the vertical length as the variation in the estimated values. The closer the plot to the diagonal, the better the prediction. Additionally, the closer the rectangle to a square, the better the prediction of the variability of the estimated values. Figure 4, in which only the mean values are plotted, shows that the plots of methods B and E are closer to the diagonal, whereas the plots of methods D and F are further away from the diagonal. The figures with rectangles show that the rectangles close to squares are more common for methods E and F. By contrast, narrow rectangles are slightly more common for method B than methods E and F. Additionally, method D has significantly more elongated rectangles. These results suggest that methods E and F were superior in predicting variation, whereas methods B and D were inferior. We compared these results with those in Table 1. We calculated *r^2^* from the mean of the measured and predicted values, thus making the metric dependent on the mean of the values. By contrast, we calculated *D*_JS_ from the normal distribution of the bone formation rate, thus making this metric dependent on both the mean and standard deviation of the bone formation rate. Because methods B and E, which had superior mean plots, also had superior *r^2^* values, *r^2^* was a suitable metric for the evaluation of mean values. *D*_JS_ was also a good metric for evaluating variation because the values of *D*_JS_ were excellent in methods E and F, where the rectangle plots were superior.

**FIGURE 5:**
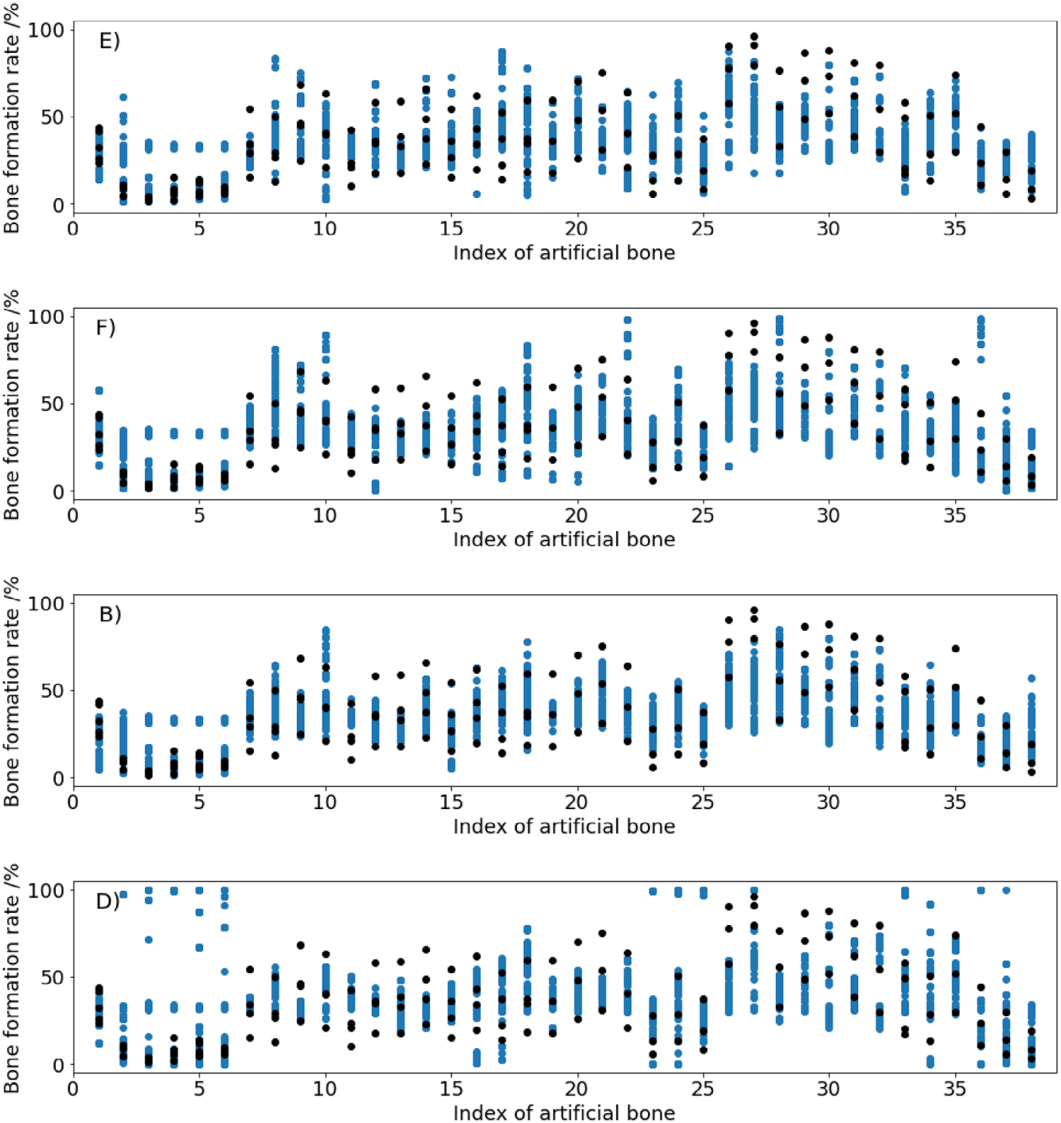
Predicted and measured bone formation rates for each artificial bone material.

**FIGURE 6:**
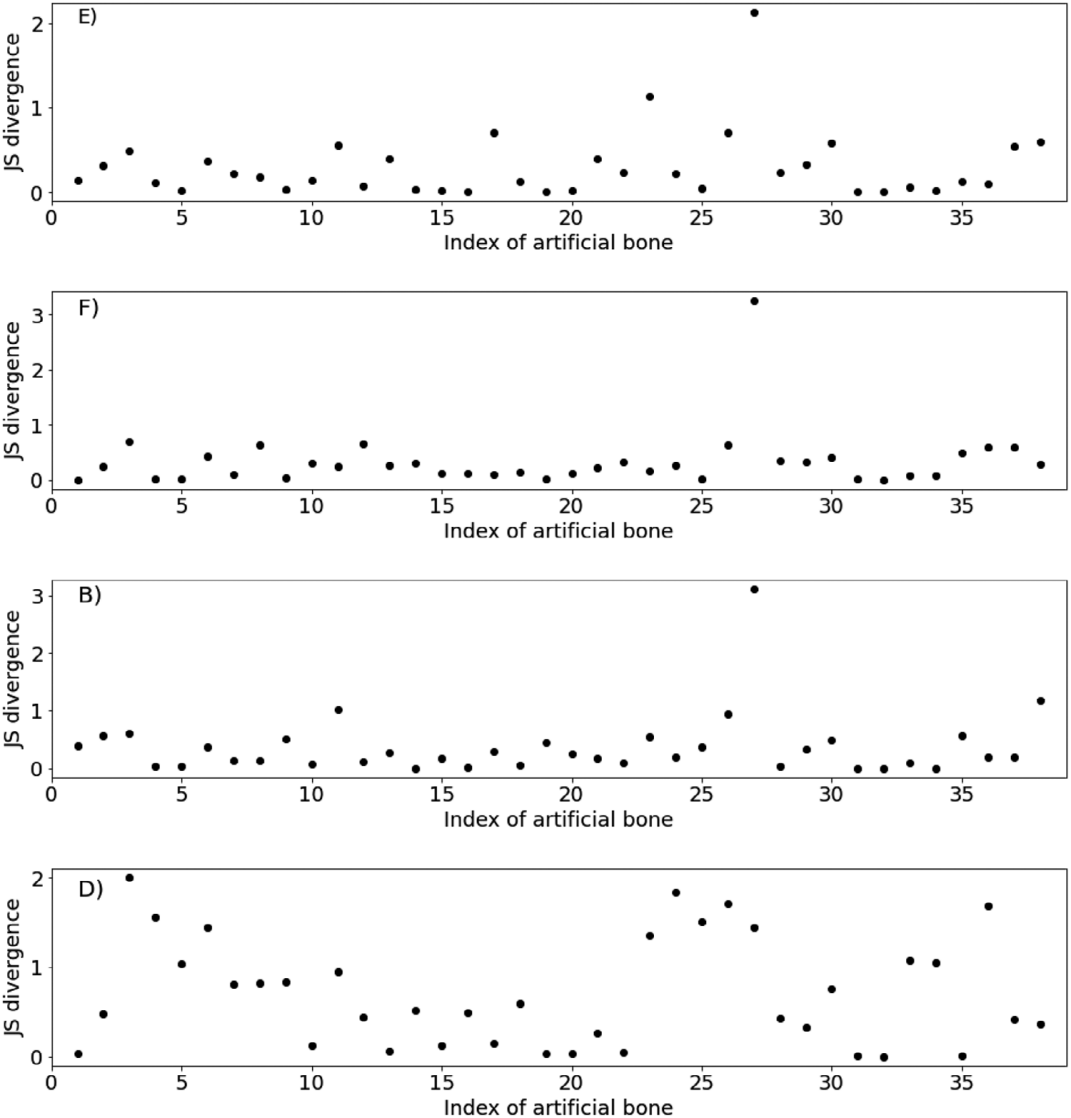
JS divergence for each artificial bone material. JS, Jensen–Shannon.

The estimated values in blue and measured values in black are plotted side by side for each artificial bone for methods E, F, B and D in Figure 5, and the other plots are in Figure S2. Additionally, *D*_JS_ for each material for methods E, F, B and D is plotted in Figure 6, and the other plots are in Figure S3. For example, for method E in Figure 5, variations between the estimated and predicted values were close for material 1, but variations were far apart for material 27. Considering the *D*_JS_ of these materials in Figure 6, the *D*_JS_ of material 1 was low and the *D*_JS_ of material 27 was high. From this result, the metric *D*_JS_ confirmed the accuracy of the prediction of the variation for each material. Figure 5 also shows that the best method, E, tended to be less accurate in predicting materials with high measured values. We replaced the standard deviations of materials with high bone formation rates to the average of the standard deviations of samples with three or more measured values because the number of measured values was small. Therefore, the fact that the actual measured variation was not the original material variation may have led to deterioration in the accuracy of the variation prediction.

## 5 Conclusions

In this study, we developed a method for constructing a machine learning model to predict an objective variable *y*, considering the variation of the experimental results in *y*. We also proposed JS divergence as a metric to evaluate the accuracy of the model for predicting variation. In a case study, we constructed a model to predict the variability of the bone formation rate, which requires long‐term measurement, using experimental conditions and experimental results, such as FT‐IR, which can be measured in a short period of time. We also verified the validity of the present index by comparing JS divergence between the measured and predicted variation. The bone formation rate in artificial bone is measured in animal experiments, which takes a long time. The degree of variability in the bone formation rate is also important in the evaluation of materials. Therefore, the ability to predict bone formation rates, including variability, based on experimental conditions, properties that can be measured in a short time, and analytical results will greatly contribute to reduction of animal testing to eliminate ethical problems and speed up development. It is also common in the chemical field that properties of materials, products, and pharmaceuticals are measured multiple times, and controlling their variability is often required. Therefore, using machine learning to predict variability will contribute to speeding up the development of materials in the chemical field.

## Conflicts of Interest

The authors declare that they have no conflict of interest.

## Supporting information



Supporting information

## Data Availability

The software that supports the findings of this study is available at https://github.com/hkaneko1985/dcekit.
