# Supplementary material for: Prediction of Bone Formation Rate of Artificial Bone With Machine Learning Models Considering the Variation of Experimental Results
Source: Anal Sci Adv. 2025 Jun 9;6(1):e70021. doi: 10.1002/ansa.70021 (PMC12147495; doi:10.1002/ansa.70021)

Table S1. Methods and combinations of variables.

| Method | FT-IR | XRD | SEM | **D_JS_** | *r^2^* |
| --- | --- | --- | --- | --- | --- |
| A | × | × | × | 0.726 | -0.496 |
| B | 〇 | × | × | 0.370 | 0.579 |
| C | × | 〇 | × | 0.383 | 0.500 |
| D | × | × | 〇 | 0.707 | -0.501 |
| E | 〇 | 〇 | × | 0.298 | 0.562 |
| F | 〇 | × | 〇 | 0.334 | 0.439 |
| G | × | 〇 | 〇 | 0.383 | 0.500 |
| H | 〇 | 〇 | 〇 | 0.361 | 0.537 |

Figure S1. Scatter plots of each average of the predicted and measured bone formation rates with rectangles showing the dispersion.


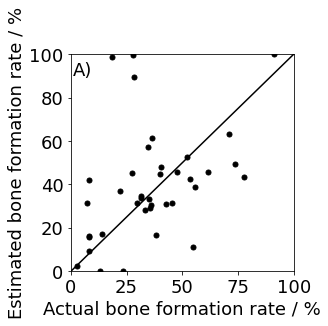

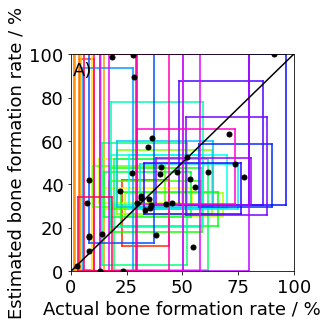


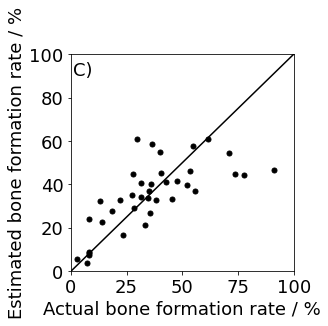

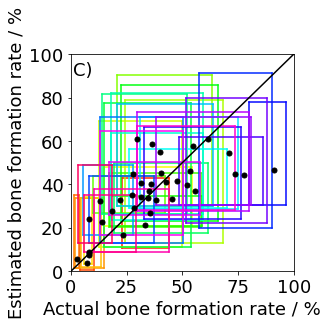


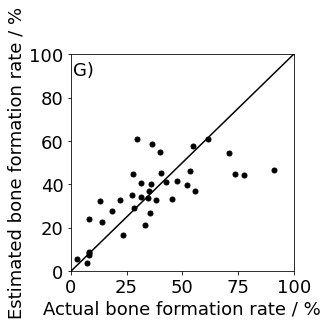

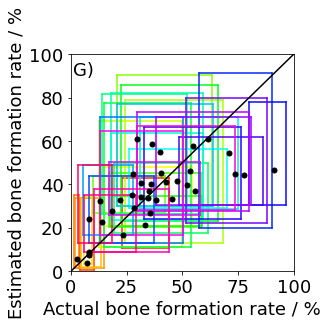


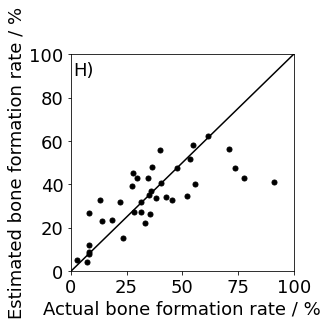

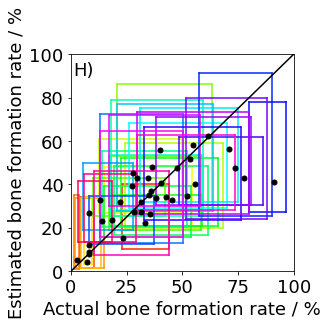


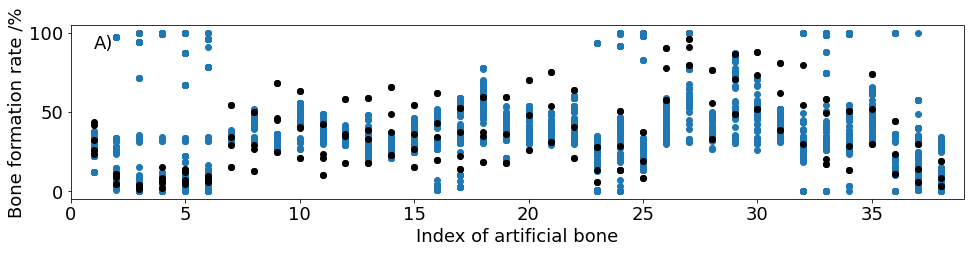


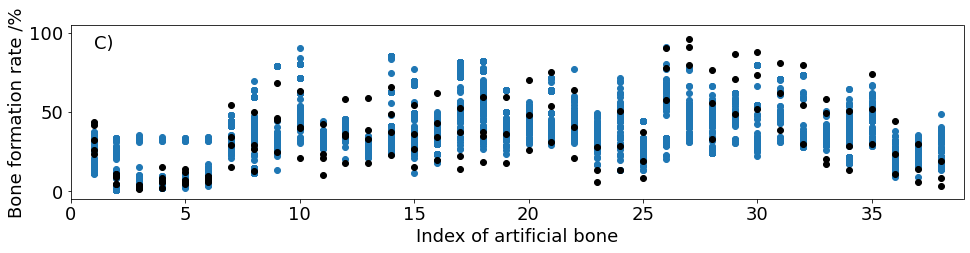


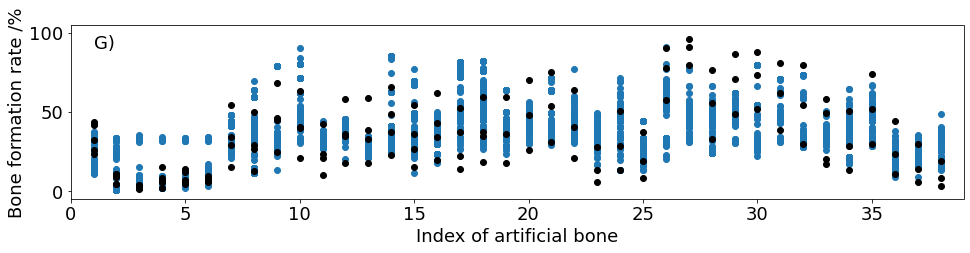


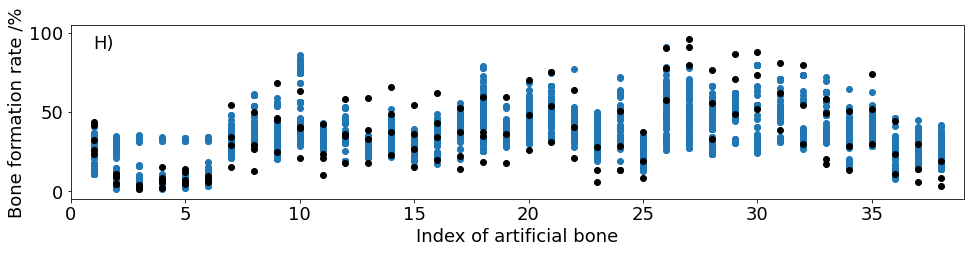


Figure S2. Predicted and measured bone formation rates for each artificial bone material.

Figure S3. JS divergence for each artificial bone material.


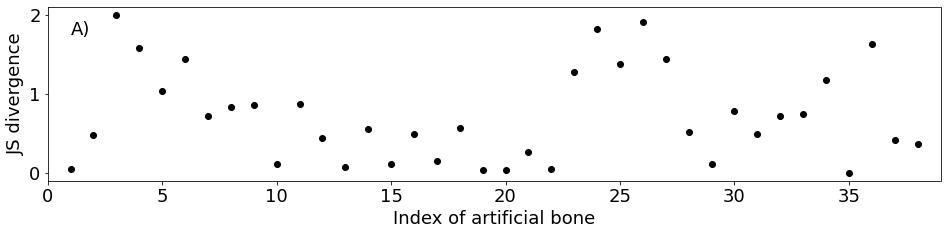


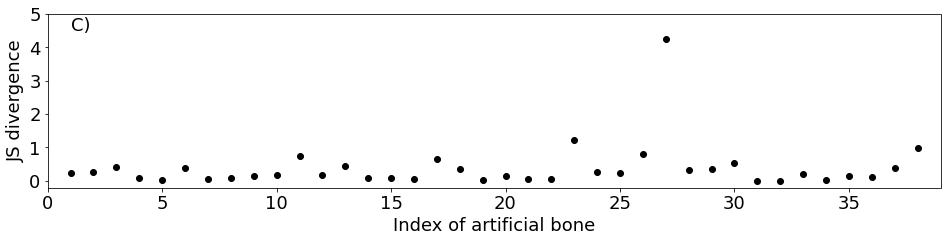


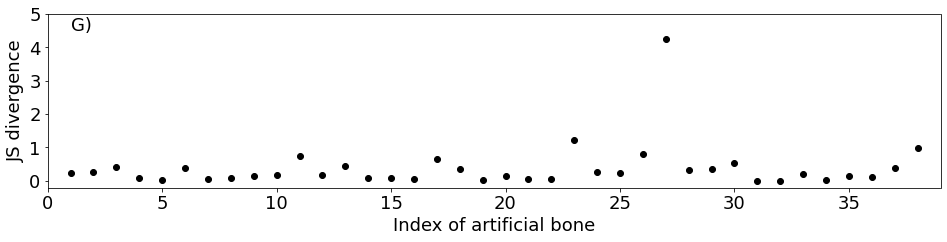


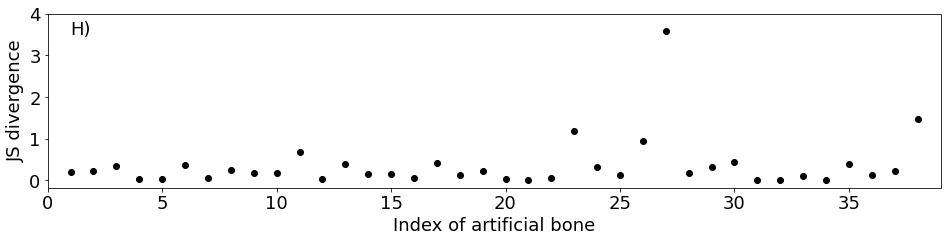

Supplement: Supplementary file 1 — Supporting information [file ANSA-6-e70021-s001.docx]
